# Antibacterial activity of bioactive compounds extracted from red kidney bean (*Phaseolus vulgaris* L.) seeds against multidrug-resistant *Enterobacterales*

**DOI:** 10.3389/fmicb.2022.1035586

**Published:** 2022-11-07

**Authors:** Azhar E. Ebrahim, Norhan K. Abd El-Aziz, Eman Y. T. Elariny, Ahmed Shindia, Ali Osman, Wael N. Hozzein, Dalal Hussien M. Alkhalifah, Dalia El-Hossary

**Affiliations:** ^1^Department of Botany and Microbiology, Faculty of Science, Zagazig University, Zagazig, Egypt; ^2^Department of Microbiology, Faculty of Veterinary Medicine, Zagazig University, Zagazig, Egypt; ^3^Department of Biochemistry, Faculty of Agriculture, Zagazig University, Zagazig, Egypt; ^4^Department of Botany and Microbiology, Faculty of Science, Beni-Suef University, Beni-Suef, Egypt; ^5^Department of Biology, College of Science, Princess Nourah Bint Abdul Rahman University, Riyadh, Saudi Arabia; ^6^Department of Medical Microbiology and Immunology, Faculty of Medicine, Zagazig University, Zagazig, Egypt

**Keywords:** *Enterobacterales*, red kidney beans, multidrug-resistant, seed storage proteins, 11S globulin, 7S globulin

## Abstract

In the present study, biologically active compounds such as phenolic-rich extract (PRE), 7S globulin (vicilin), and 11S globulin (legumin) from red kidney bean (*Phaseolus vulgaris* L.) seeds were extracted and evaluated as antibacterial agents against multidrug-resistant (MDR) *Enterobacterales* isolated from both animal and human sources. The overall occurrence rate of *Enterobacterales* was 43.6%, which significantly differed between animal (38.75%) and human (56.67%) sources. Antimicrobial susceptibility testing revealed that *Enterobacterales* isolates exhibited full resistance (100%) to amoxicillin-clavulanic acid, followed by ampicillin (75.44%), erythromycin (71.93%), cefoxitin (70.18%), amoxicillin (66.66%), ceftriaxone (64.91%), and trimethoprim/sulfamethoxazole (56.14%). Worthy of note, 97.92% of *Enterobacterales* isolates were MDR. The total phenolic contents (TPC; 53 ± 2 mg GAE g-1) and total flavonoid contents (TFC; 26 ± 1 mg QE g-1) were recorded. The major phenolic and flavonoid components were catechol (17.63 μg/mL) and hesperidin (11.37 μg/mL), respectively. Sodium dodecyl sulfate-polyacrylamide gel electrophoresis (SDS-PAGE) was performed to detect the 7S and 11S globulin‘s molecular mass. The data revealed that red kidney bean protein isolate (KPI) includes two major portions: 7S and 11S globulins. The bioactive compounds of *Phaseolus vulgaris* were investigated for their antibacterial activities against *Enterobacterales* for the first time. The protein component (MIC = 0.125 – 2 μg/mL; 53.85%) and its 7S and 11S globulin subunits (MIC = 0.5 – 2 μg/mL; 30.77% each) were the most potent extracts, whereas the methanolic extract was the least effective one (MIC = 2 μg/mL; 15.38%). The results displayed the potential of protein bioactive compounds as a hopeful candidate for enhancing future medication plans for the treatment of *Enterobacterales* originating from animal and human sources.

## Introduction

*Enterobacterales* are the most challenging bacterial contaminant in raw and processed meat products worldwide, with a prominence of *Escherichia coli* (*E. coli*), *Salmonella, Proteus*, and *Klebsiella* species in food poisoning cases (Mohammed et al., 2014). The risk of this group comes from the high contamination level of examined meat at slaughterhouses, which begins from skinning, evisceration, washing, handling, and transportation that may cause foodborne illness (Duffy et al., 2003; Severgnini et al., 2011).

Antimicrobial resistance is a serious issue. It has evolved into a significant public health concern of the 21st century. The National Antimicrobial Resistance Monitoring System (NARMS) surveys indicated that retail meat is frequently contaminated with multidrug-resistant (MDR) bacteria such as *Staphylococcus aureus, Salmonella* species, and *E. coli* (Food and Drug Administration, 2007). Antimicrobial resistance and MDR bacterial strains are frequent in hospitals and might impair worldwide infectious disease management (Terreni et al., 2021).

Nowadays, increased mortalities per year are attributed to nosocomial infections with antimicrobial resistance; future years are expected to be considerably worse, as revealed recently (Zurabov and Zhilenkov, 2021). Hence, searching for novel antimicrobials derived from natural sources is an essential component of contemporary medicine to combat the socio-economic influence and health impact caused by MDR bacteria (Bakal et al., 2017; Khalid et al., 2021; Sabry et al., 2022).

A wide variety of secondary metabolites are produced by plants, many of which exhibit antibacterial properties against some pathogenic bacteria linked to gastrointestinal illnesses. Some of these components are constitutive, exist in healthy plants in their physiologically active forms, and exhibit chemotherapeutic or chemoprophylactic characteristics against a variety of enteric infectious illnesses (Godstime and Akpotu, 2014).

The proteins in legume seeds as beans (*Pharsalus* species) and peas (*Pisum sativum*) are about 20%, whereas in lupin (*Lupinus* species) and soybean (*Glycine max*) are up to 40% (Đorpevic’, 2009). The globulins predominate as storage proteins and typically account for 50–90 percent of the protein content in legume seeds. Some of these proteins were recognized to have important pharmacological and therapeutic values (Scarafoni et al., 2007).

Four out of five persons turn to conventional medicine for their healthcare necessities (Kebede et al., 2021). However, medicinal plants are equipped with a variety of bioactive chemicals to combat multidrug resistance (Kebede et al., 2021). Beans proteins such as lectins, arcelins, and protease and amylase inhibitors were proven to have antimicrobial properties (Ma et al., 2009). Roy et al. (2020) documented that dark red kidney bean protein hydrolyzates may be used as auspicious antimicrobial and antioxidant candidates. Previous studies described the significance of 7S and 11S globulins from different sources as antimicrobial agents (Sitohy et al., 2012; Osman et al., 2013; Mahgoub et al., 2013, 2016; Abbas et al., 2020; Atallah et al., 2021). To further enrich the library of natural antibacterial agents, the objective of the work was to investigate the antibacterial activities of 11S globulins, 7S globulins, and phenolic-rich extracts isolated from red kidney bean seeds against MDR *Enterobacterales* isolated from animal and human sources.

## Materials and methods

### Sampling

From August 2020 to June 2021, 110 samples were collected from both animal (*n* = 80) and human (*n* = 30) sources. Animal samples, including raw milk (*n* = 25), milk products (*n* = 17), fresh meat (*n* = 12), and meat products (*n* = 26), were drawn randomly from various supermarkets in Fakous City, Sharkia Governorate, Egypt. In addition, human stools (*n* = 30) were obtained from attending patients at various laboratories and hospitals in Fakous City, Sharkia Governorate. The samples were put into sterile containers aseptically, stored in an ice bag, and transferred immediately to the bacteriology laboratory for their examination upon arrival. The declaration of Helsinki, the World Medical Association’s Code of Ethics, was followed during the study’s execution. The patients provided informed written consent to take part in this investigation.

### Isolation and identification of *Enterobacterales*

A loopful from each supplemented broth was grown on an agar medium that was selective for all predictable bacteria; MacConkey‘s agar (Oxoid, Cambridge, UK) for coliforms, Eosin Methylene Blue agar (EMB; Oxoid, Cambridge, UK) for *E. coli*, HiCrome Klebsiella selective agar (Himedia, Thane West, India) for *Klebsiella* species and MacConkey‘s agar without salt (Oxoid, Cambridge, UK) for *Proteus* species. To isolate *Salmonella*, one gram of each sample was inoculated into 9 mL of buffered peptone water (BPW; Oxoid, Cambridge, UK), followed by incubation for 18 h at 37°C. One-tenth mL of the BPW was injected into 10 mL of Rappaport Vassiliadis soy broth (RV; Oxoid, Cambridge, UK), then incubation was done for 24 h at 42°C. Selective plating was applied using Xylose Lysine Deoxycholate agar (XLD; Sigma-Aldrich, St. Louis, USA). Gram stain was used to identify a suspected colony of each bacterium, then conventional biochemical assays (Faddin, 2000) were performed to identify each bacterial species. Biochemically identified *E. coli* isolates were serotyped by the rapid agglutination approach using specific antisera (Denka Seiken Co., Tokyo, Japan). Molecular confirmation of Gram-negative isolates was done by polymerase chain reaction (PCR) utilizing the species-specific primer pairs indicated in Supplementary Table 1 (Brisse and Verhoef, 2001; Murugkar et al., 2003; Kumar et al., 2008; Turton et al., 2010; Bi et al., 2013; Litty et al., 2013; Zhang et al., 2013; Halimi et al., 2014). PCR amplimers were visualized by a U.V. transilluminator (Sigma-Aldrich, St. Louis, USA) after electrophoresis for 30 min on a 2% agarose gel incorporated with 0.5 μg/mL ethidium bromide.

### Antimicrobial susceptibility testing of *Enterobacterales*

The antimicrobial susceptibilities of *Enterobacterales* against 17 antimicrobial agents were detected by adopting the Clinical and Laboratory Standards Institute [CLSI] recommendations (Clinical and Laboratory Standards Institute [CLSI], 2020). The examined antimicrobials (Oxoid, Cambridge, UK) were penicillins [amoxicillin (25 μg), amoxicillin-clavulanic acid (20/10 μg), ampicillin (10 μg), ampicillin-sulbactam (10/10 mg)], cephalosporins [cefoxitin (30 μg), ceftriaxone (30 μg), cefepime (30 μg)], carbapenems [imipenem (10 μg)], fluoroquinolones [ciprofloxacin (5 μg)], aminoglycosides [gentamicin (10 μg), amikacin (30 μg)], sulfonamides [trimethoprim-sulfamethoxazole (1.25/23.75 μg)], macrolides [erythromycin (15 μg)], tetracyclines [doxycycline (30 μg)], chloramphenicol [chloramphenicol (30 μg)], monobactam [aztreonam (10 μg)], and glycylcycline [tigecycline (15 μg)]. The multiple antibiotic resistance (MAR) indices were assessed according to Tambekar et al. (2006), and the MDR isolates were recorded following Magiorakos et al. (2012).

### Red kidney bean (*Phaseolus vulgaris* L.) seeds

Red kidney bean seeds were obtained from a local outlet in Zagazig City, Sharkia Governorate, Egypt. The seeds were manually cleaned, ground, and defatted using a Soxhlet apparatus (Prosperity Biotech Co., Ltd., Shandong, China) with hexane for 6–8 h. The solvent was vaporized using a rotary evaporator (VWR International GmbH, Darmstadt, Germany), and the defatted dried meal was preserved at 4°C until analysis.

### Preparation and characterization of phenolic-rich kidney bean extract

The phenolic-rich red kidney bean extract (PRKE) was prepared as designated elsewhere (Abdel-Shafi et al., 2019a; Omar et al., 2020). Briefly, defatted red kidney bean flour (100 g) was extracted for 2 h in darkness at room temperature with 70% v/v aqueous methanol (1 L). Centrifugation of the sample was done for 15 min at 10,000 × *g* to recover the supernatant. Removal of the methanol from the extract was performed by vacuum evaporation in a BüCHI-water bath B-480 evaporator (Buchi, Switzerland) at 45°C followed by a freeze-dryer lyophilization (Thermo-electron Corporation–Heto power dry LL 300 Freeze drier, Bossgoo, China).

The total phenolic contents (TPCs) of the PRKE (1,000 μg/mL) were calculated by the Folin–Ciocalteu assay (Singleton et al., 1999). To create a standard curve, various concentrations of gallic acid (10–2000 g/mL) were dissolved in distilled water. The gallic acid calibration equation was as follows: *y* = 0.001x + 0.0563 (R2 = 0.9792), where *y* and *x* stand for the gallic acid absorbance and concentration expressed in μg/mL, respectively. The reaction mixture [1 mL from the standard solution or extract + 3 mL Folin-Ciocalteu diluted with distilled water (1:10, V/V) + 2 mL Na_2_Co_3_ 7.5%] was agitated for one min, then kept at room temperature for 30 min in the darkness. The mixture absorbance was determined at 765 nm using a JANEWAY spectrophotometer (6405 UV/Vis, Missouri, UK). Gallic acid equivalents (GAE) per gram of extract were used to measure the sample’s ability to reduce the Folin-Ciocalteu reagent.

Total flavonoids of the PRKE (1,000 μg/mL) were approximated following Ordonez et al. (2006) a protocol as described previously (Abdel-Shafi et al., 2019b). Quercetin was dissolved in ethyl alcohol at various concentrations (10–1000 μg/mL) to get a standard curve. Total flavonoid contents (TFCs) are indicated as quercetin equivalent (QE). The later was computed using the subsequent calibration curve: *y* = 0.0012x + 0.008 (R2 = 0.944), where y means the absorbance and *x* refers to the concentration of quercetin in μg/mL. The reaction mixture involved 500 μL from the standard solution or the extract plus 1,000 μL aliquot of 20 g/L AlCl3 ethanol. The absorbance at 420 nm was recorded using a spectrophotometer (JENWAY, Missouri, UK).

High-performance liquid chromatography (HPLC)-(Agilent 1100) analysis is comprised of two liquid chromatography (LC) pumps, a C18 column (5 μm particle size; 125 mm × 4.60 mm), and a UV/Vis detector. The Agilent ChemStation Phenolic acids could analyze the chromatograms, which were then separated by engaging a gradient mobile phase of two solvents; methanol (solvent A) and acetic acid in water (solvent B; 1:25). Solvent B was used at 100% concentration to start the gradient program and remained there for the first 3 min. Thereafter, 50% eluent A was added for the next 5 min. The eluent A`s concentration was then raised to 80% for the following 2 min, then decreased to 50% once more for the subsequent 5 min, then detection of the wavelength was set at 250 nm.

### Red kidney bean protein and its subunits isolation and characterization

Defatted red kidney bean flour (5%) dispersions in water were accustomed to pH 9 at room temperature with 0.1 N NaOH, centrifuged for 15 min at 2,000 × *g* after being agitated for an hour. To improve the yield, repeated isolation and centrifugation operations on the residue were performed. To precipitate the protein, the extracts were mixed and the pH was accustomed to 4.5 using 1N HCl. Thereafter, the proteins were extracted by centrifugation for 15 min at 2,000 × *g*. Decantation of the supernatant was done. After washing in distilled water, the crude protein was dispersed in a bit of water with a pH of 7.5, dialyzed overnight, and lyophilized (Johnson and Brekke, 1983). With minor changes, the 7S and 11S globulin subunits were extracted from the lupine seed‘s defatted powder using the procedures reported earlier (Abdel-Shafi et al., 2019a).

### Sodium dodecyl sulfate-polyacrylamide gel electrophoresis

The defatted red kidney bean flour and crude protein isolate were melted in 1 mL of 10% SDS, 100 μL β-mercaptoethanol, and 20 mg of kidney bean protein. The mixture was then vortexed occasionally for 15 min. To separate the extract, centrifugation was done at 10,000 × *g* for 5 min. Twenty μL of extracted proteins were mixed with 20 μL of SDS-loading sample buffer (4% SDS, 3% β-mercaptoethanol, 20% glycerol, Tris-HCl 50 mM pH 6.8, and bromophenol blue traces), then heated for five min at 96°C. Aliquots of 10 μL of protein per lane were electrophoresed and analyzed by sodium dodecyl sulfate-polyacrylamide gel electrophoresis (SDS-PAGE) (Laemmli, 1970).

### Urea-polyacrylamide gel electrophoresis

Lyophilized red kidney bean protein, 7S, and 11S globulins from red kidney bean seeds (10 mg/mL) were dispersed in a 6.8 pH buffer comprising bromophenol blue traces, 50% glycerol, and 0.25 M Tris-base. Centrifugation of samples (15,000 × *g* for 5 min) was applied at 20°C. Urea-PAGE analysis of the supernatants (10 μL of protein per lane) was performed using stacking (3%) and resolving (12%) gels (Evans and Williams, 1980).

### The pH-solubility curve

Lyophilized red kidney bean protein, 7S, and 11S globulins from red kidney bean seeds were tested for pH-solubility curves in the pH 2–10 range (Chobert et al., 1991). Since the pH corresponds to the least solubility of proteins, the isoelectric points were calculated.

### Fourier transform infrared spectroscopy

The potassium bromide (KBr) pellet method was followed to prepare the protein samples (Souillac et al., 2002). An Fourier transform infrared (FTIR) spectrometer (Thermo Scientific, Waltham, MA, USA) was used to measure infrared spectra at a temperature of 25°C. Thereafter, 256 interfero grams were obtained for each spectrum with a resolution of 4 cm^–1^ with 64 scans and a 2 cm^–1^ interval from the 4,000 to 400 cm^–1^ regions. The system was incessantly purged with dry air. Savitsky-Golay derivative function soft was used to get second derivation spectra. The areas under the bands associated with a particular substructure were manually computed from the infrared second derivative amide spectra to determine their relative amounts.

### Antibacterial activity of biologically active compounds isolated from red kidney bean seeds

The antibacterial activities of red kidney beans were evaluated against MDR *Enterobacterales*. The agar well diffusion assay was performed (Valgas et al., 2007), and the bacterial isolates with inhibition zones diameters less than or equal to 8 mm were deemed susceptible (Choi et al., 2016). The broth microdilution technique was adopted to determine the minimum inhibitory concentrations (MICs) of the studied antimicrobials (Rankin, 2005).

### Statistical analysis

All the study experiments were performed in triplicates. Data were edited in Microsoft Excel (Microsoft Corporation, Redmond, WA, USA). Fisher exact test was applied to detect the significant differences in microbial presence in different animal and human sources. Kruskal–Wallis Test was used to compare between the newly tested antimicrobial agents per concentrate (25, 50, 75, and 100%). Figures were fitted by the Graph-Pad Prism software 5.0 (Graph Pad, USA). A *p-value* of <0.05 is considered statistically different.

## Results

### Bacteriological analysis and PCR identification of *Enterobacterales*

In this study, the bacteriological analysis revealed that *Enterobacterales* isolates were detected in 48 out of 110 examined samples from animal and human sources with a percentage of 43.63%. Microscopical examination of characteristic colonies stained with Gram stain revealed Gram-negative, medium-sized bacilli, non-spore-forming and arranged single, pairs, and in groups. *Klebsiella* isolates showed mucoid colonies with purple magenta color on the Klebsiella selective agar, large mucoid pink to purple colonies on EMB, and pink mucoid colonies (rapid lactose fermenters) on MacConkey’s agar media. Green metallic sheen colonies on EMB media were characteristic of *E. coli* and *Citrobacter freundii*, which were identified using a citrate test that exhibited a negative reaction with *E. coli*. On XLD agar media, slight transparent reddish-colored colonies with a black center were characteristic of *Salmonella* or *Proteus* species. Both showed red slant, yellow butt with H_2_S production on TSI agar, which were differentiated using a urease test; *Salmonella* species is urease negative (yellow color), while *Proteus* is a rapid urease producer (pink color). The O-H serotyping of cultural and biochemically positive *E. coli* (*n* = 23) revealed 7 serotypes including O26:H11 (*n* = 5), O111:H2 (*n* = 3) O51:H49, O146:H21 (*n* = 2 each) and O28:H28 (*n* = 1) for human isolates, O146:H21 (*n* = 3) and O26:H11, O86:H21 (*n* = 2 each) for milk isolates and O26:H11 (*n* = 2) and O55:H7 (*n* = 1) for meat isolates.

Suspected *Enterobacterales* isolates were further confirmed by PCR. Twenty-three isolates were positive for the *uidA* gene giving characteristic bands at 530 bp to be assured as *E. coli* (Supplementary Figures 1A,B). Seventeen isolates were identified as *K. pneumoniae* following the PCR-based genus (*gyrA*) and species-specific identification (*K. pneumoniae 16S-23ITS*) genes giving characteristic bands at 441 and 130 bp, respectively (Supplementary Figures 2A,B). Whilst, six isolates had the *ureR* gene supposing them as *Proteus mirabilis* at characteristic bands of 101 bp (Supplementary Figures 3A,B). Eleven *Enterobacterales* isolates had the genus-specific *invA* gene of *Salmonella* species (248 bp product size), ten of them were identified as *Salmonella* Typhimurium (*fliC* gene; 613 bp amplicon), and one was *Salmonella* Enteritidis (*sefC* gene; 1104 bp) (Supplementary Figures 4A–C).

### Occurrence of *Enterobacterales* in animal and human sources

As documented in Table 1, the occurrence of *Enterobacterales* was 43.6% (48/110), which significantly differed between animal (31/80, 38.75%) and human (17/30, 56.67%) sources. A relatively high occurrence rate of *Enterobacterales* was reported in raw milk (15/25, 60%) followed by fresh meat (7/12, 58.3%), milk products (5/17, 29.4%), and meat products (4/26, 15.3%).

**TABLE 1:** Occurrence of *Enterobacterales* in examined milk and milk products, meat and meat products, and human stools.

*Enterobacterales* Species	Samples of animal origin (*n* = 80)											Human stool (*n* = 30)	*P*-value
	Raw milk	Milk products (*n* = 17)			Fresh meat and meat products (*n* = 38)						Total		
	RM (*n* = 25)	RC (*n* = 7)	MC (*n* = 5)	KC (*n* = 5)	FM (*n* = 12)	CM (*n* = 7)	S (*n* = 6)	B (*n* = 5)	MM (*n* = 5)	N (*n* = 3)			
*E. coli*	1 (4)	1 (14.3)	2 (40)	0 (0)	2 (16.6)	0 (0)	0 (0)	0 (0)	1 (20)	0 (0)	7 (8.7)	7 (23.3)	0.6726
*K*. *pneumonia*	11 (44.0)	0 (0)	0 (0)	0 (0)	2 (16.6)	0 (0)	0 (0)	0 (0)	1 (20)	0 (0)	14 (17.5)	0 (0)	0.0104
*Salmonella*	0 (0)	1 (14.2)	0 (0)	0 (0)	0 (0)	0 (0)	0 (0)	1 (20)	1 (20)	0 (0)	3 (3.7)	3 (10)	0.2018
*P. mirabilis*	0 (0)	0 (0)	0 (0)	1 (20)	3 (25)	0 (0)	0 (0)	0 (0)	0 (0)	0 (0)	4 (5.0)	1 (3.3)	0.8377
*E. coli* + *P. mirabilis*	0 (0)	0 (0)	0 (0)	0 (0)	0 (0)	0 (0)	0 (0)	0 (0)	0 (0)	0 (0)	0 (0)	1 (3.3)	0.2727
*E. coli* + *Salmonella*	0 (0)	0 (0)	0 (0)	0 (0)	0 (0)	0 (0)	0 (0)	0 (0)	0 (0)	0 (0)	0 (0)	5 (16.6)	0.0012
*E. coli* + *K*. *pneumoniae*	3 (12)	0 (0)	0 (0)	0 (0)	0 (0)	0 (0)	0 (0)	0 (0)	0 (0)	0 (0)	3 (3.7)	0 (0)	0.3807
*p*-value	0.0001	0.0001	0.0001	0.0001	0.0001	ND	ND	0.0005	0.0021	ND	0.0001	0.0001	0.0001

RM, Raw Milk; RC, Romy Cheese; MC, Mozzarella Cheese; KC, Karish Cheese; FM, Fresh Meat; CM, Corned Meat; S, Sausage; B, Burger; MM, Minced Meat; N, Nugget. Data are represented by frequencies (%); ND, non–determined; *p*-values were calculated according to Fisher‘s exact test.

Worthy of note, the highest prevalence of *Enterobacterales* was detected for each of *E. coli* and *K. pneumoniae* (14/48, 29.17%), followed by *Salmonella* (6/48, 12.5), and *P. mirabilis* (5/48, 10.42) species. Mixed bacterial contamination was reported for *E. coli* + *Salmonella* (5/48, 10.42%), *E. coli* + *K. pneumoniae* (3/48, 6.25%), and *E. coli* + *P. mirabilis* (1/48, 2.08%). Statistical analysis revealed significant differences (*P* < 0.05) in the occurrence of *K. pneumoniae* or mixed infection of *E. coli* with *Salmonella* in animal and human samples. However, the existence of various *Enterobacterales* among each animal and human sources showed highly significant variations (*P* = 0.0001).

### Antibiogram of *Enterobacterales* isolates

**FIGURE 1:**
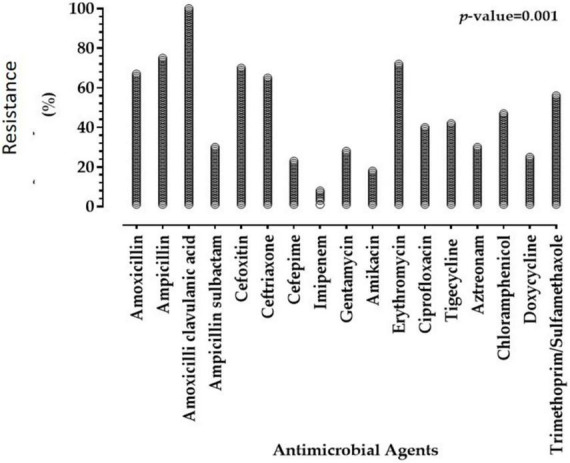
Resistance of *Enterobacterales* isolates of animal and human origins to antimicrobial agents.

Antimicrobial susceptibilities of 57 *Enterobacterales* isolates to 17 antimicrobials of diverse classes are summarized in Tables 2, 3, and Figure 1. The results declared that all *Enterobacterales* isolates of animal and human origins showed resistance to amoxicillin-clavulanate (100%), followed by ampicillin (75.44%), erythromycin (71.93%), cefoxitin (70.18%), amoxicillin (66.66%), ceftriaxone (64.91%), and trimethoprim/sulfamethoxazole (56.14). However, a moderate sensitivity level was observed for chloramphenicol (47.37%), followed by ciprofloxacin and tigecycline (40.35 and 42.11%, respectively). *Enterobacterales* showed a high sensitivity level for imipenem (91.23%). MAR indices of most *Enterobacterales* isolates were higher than 0.2 (0.235–1.0), except one showed a MAR index of 0.176. Interestingly, 97.92% of *Enterobacterales* isolates were categorized as MDR. Statistical analysis showed significant variations in the antimicrobial resistance among *Enterobacterales* species for amoxicillin, ampicillin-sulbactam, cefepime, tigecycline, aztreonam, and doxycycline (*P* < 0.05).

**TABLE 2:** Antimicrobial resistance of *Enterobacterales* isolates (*n* = 57) recovered from human and animal origins.

Antimicrobial agent	*Enterobacterales* isolates					MAR index	*P*-value
	*E. coli* (*n* = 23)	*K. pneumoniae* (*n* = 17)	*Salmonella* (*n* = 11)	*P. mirabilis* (*n* = 6)	Total *Enterobacterales* (*n* = 57)		
Amoxicillin (AX)	20 (86.9)	11 (64.7)	11 (100)	6 (100)	38 (66.66)	0.039	0.024
Ampicillin (AMP)	20 (86.9)	16 (94.1)	10 (90.9)	4 (66.6)	43 (75.44)	0.044	0.352
Amoxicillin clavulanic acid (AMC)	23 (100)	17 (100)	11 (100)	6 (100)	57 (100)	0.058	1.00
Ampicillin sulbactam (SAM)	2 (8.6)	11 (64.7)	3 (27.2)	1 (16.6)	17 (29.82)	0.017	0.001
Cefoxitin (FOX)	18 (78.2)	14 (82.3)	8 (72.7)	6 (100)	40 (70.18)	0.041	0.572
Ceftriaxone (CRO)	16 (69.5)	14 (82.3)	8 (72.7)	3 (50.0)	37 (64.91)	0.038	0.491
Cefepime (FEB)	10 (43.4)	0 (0.00)	5 (45.4)	2 (33.3)	13 (22.81)	0.013	0.014
Imipenem (IMP)	5 (21.7)	0 (0.00)	0 (0)	1 (16.6)	5 (8.77)	0.005	0.085
Gentamycin (CN)	6 (26.0)	3 (17.6)	5 (45.4)	2 (33.3)	16 (28.07)	0.016	0.442
Amikacin (AK)	6 (26.0)	4 (23.5)	1 (9.0)	0 (0)	10 (17.54)	0.010	0.384
Erythromycin (E)	20 (86.9)	14 (82.3)	10 (90.9)	5 (83.3)	41 (71.93)	0.042	0.927
Ciprofloxacin (CIP)	8 (34.7)	10 (58.8)	7 (63.6)	3 (50.0)	23 (40.35)	0.023	0.325
Tigecycline (TGC)	5 (21.7)	13 (76.4)	3 (27.2)	3 (50.0)	24 (42.11)	0.024	0.004
Aztreonam (ATM)	8 (34.7)	0 (0.00)	6 (54.5)	4 (66.6)	17 (29.82)	0.017	0.002
Chloramphenicol (C)	9 (39.1)	11 (64.7)	6 (54.5)	3 (50.0)	27 (47.37)	0.027	0.452
Doxycycline (DO)	8 (34.7)	3 (17.6)	1 (90.9)	2 (33.3)	14 (24.56)	0.014	0.048
Trimethoprim/sulfamethoxazole (SXT)	11 (47.8)	13 (76.4)	7 (63.6)	5 (83.3)	32 (56.14)	0.033	0.197
*P-value*	0.001	0.001	0.001	0.002	0.001	—-	—-

MAR, multiple antibiotic resistance.

**TABLE 3:** Antimicrobial resistance patterns of 57 *Enterobacterales isolates* recovered from 48 samples of animal and human origins.

Sample no.	*Enterobacterales*isolates (*n* = 57)	Source	Antimicrobial resistance pattern
1	*K. pneumoniae*	Raw milk	AMP, AMC, CN, TGC, SAM, CRO, E, FOX, CIP, C, DO, SXT, AX
2	*K. pneumoniae*	Raw milk	AMP, AMC, TGC, SAM, CRO, E, FOX, CIP, C, DO, SXT, AX
3	*K. pneumoniae*	Raw milk	AMP, AMC, TGC, SAM, CRO, E, FOX, CIP, C, SXT, AX
4	*K. pneumoniae*	Raw milk	AMP, AMC, SAM, CRO, E, FOX, CIP, C, SXT, AX
5	*K. pneumoniae*	Raw milk	AMP, AMC, TGC, SAM, CRO, E, FOX, CIP, C, DO, SXT, AX
6	*K. pneumoniae*	Fresh meat	AMP, AMC, CN, TGC, CRO, E, SXT
7	*K. pneumoniae*	Fresh meat	AMP, AMC, CN, TGC, AK, CRO, E, SXT
8	*K. pneumoniae*	Raw milk	AMP, AMC, TGC, SAM, CRO, E, FOX, CIP, C, SXT, AX
9	*S.*Typhimurium	Meat product (Minced)	AMP, AMC, CN, TGC, ATM, CRO, E, C, AX
10	*K. pneumoniae*	Raw milk	AMP, AMC, TGC, AK, CRO, E, SXT, AX
11	*K. pneumoniae*	Raw milk	AMP, AMC, TGC, E, FOX, AX
	*E. coli*		AMP, AMC, TGC, E, FOX, AX
12	*S.*Enteritidis	Meat product (Burger)	AMP, AMC, CN, TGC, ATM, FEB, CRO, E, FOX, CIP, C, AX
13	*E. coli*	Milk product (Romi)	AMP, AMC, IPM, FEB, CRO, E, CIP, C, DO, SXT, AX
14	*P. mirabilis*	Milk product (Karish)	AMP, AMC, TGC, FOX, SXT, AX
15	*K. pneumoniae*	Raw milk	AMP, AMC, TGC, SAM, CRO, FOX, CIP, C, DO, SXT
16	*P. mirabilis*	Fresh meat	AMP, AMC, CN, TGC, AK, IPM, SAM, ATM, FEB, CRO, E, FOX, CIP, C, DO, SXT, AX
17	*K. pneumoniae*	Raw milk	AMP, AMC, SAM, CRO, E, FOX, C, AX
	*E. coli*		AMP, AMC, SAM, CRO, E, FOX, C, AX
18	*K. pneumoniae*	Raw milk	AMP, AMC, TGC, SAM, CRO, E, FOX, CIP, C, SXT
19	*K. pneumoniae*	Raw milk	AMP, AMC, TGC, SAM, CRO, E, FOX, CIP, C, SXT
20	*E. coli*	Fresh meat	AMC, AK, FOX
21	*E. coli*	Fresh meat	AMC, AK, CRO, FOX, C
22	*K. pneumoniae*	Raw milk	AMP, AMC, AK, SAM, CRO, FOX, CIP, C, SXT
23	*K. pneumoniae*	Meat product (Minced)	AMP, AMC, TGC, AK, E, FOX, AX
24	*S.*Typhimurium	Milk product (Romi)	AMP, AMC, ATM, FOX, CIP, AX
25	*P. mirabilis*	Fresh meat	AMP, AMC, TGC, E, FOX, AX
26	*E. coli*	Milk product (Mozzarella)	AMP, AMC, TGC, E, FOX, AX
27	*P. mirabilis*	Fresh meat	AMC, CN, ATM, CRO, E, FOX, C, AX
28	*K. pneumoniae*	Raw milk	AMP, AMC, E, FOX
	*E. coli*		AMP, AMC, CRO, E, FOX, C, DO, AX
29	*E. coli*	Meat product (Minced)	AMP, AMC, TGC, CRO, E, FOX, DO, AX
30	*E. coli*	Raw milk	AMP, AMC, ATM, AX
31	*E. coli*	Milk product (Mozzarella)	AMP, AMC, CN, TGC, AK, IPM, SAM, ATM, FEB, CRO, E, FOX, CIP, SXT
32	*S.*Typhimurium	Human stool	AMC, SAM, E, FOX, CIP, SXT, AX
	*E. coli*		AMP, AMC, CN, TGC, AK, IPM, ATM, FEB, CRO, E, FOX, CIP, C, DO, SXT, AX
33	*S.*Typhimurium	Human stool	AMP, AMC, FEB, CRO, E, FOX, AX
	*E. coli*		AMP, AMC, FEB, CRO, E, FOX, SXT, AX
34	*S.*Typhimurium	Human stool	AMP, AMC, SAM, E, FOX, C, SXT, AX
	*E. coli*		AMP, AMC, ATM, CRO, E, FOX, C, SXT, AX
35	*E. coli*	Human stool	AMP, AMC, AK, IPM, FEB, CRO, E, CIP, DO, SXT, AX
36	*S.*Typhimurium	Human stool	AMP, AMC, ATM, CRO, E, FOX, C, SXT, AX
37	*E. coli*	Human stool	AMC, CN, ATM, CRO, E, FOX, AX
38	*E. coli*	Human stool	AMC, E, FOX, AX
39	*P. mirabilis*	Human stool	AMP, AMC, CN, SAM, ATM, CRO, E, FOX, CIP, C, DO, SXT, AX
40	*E. coli*	Human stool	AMP, AMC, CN, FEB, CRO, E, C, SXT, AX
41	*E. coli*	Human stool	AMP, AMC, E, FOX, CIP, SXT, AX
42	*E. coli*	Human stool	AMP, AMC, E, FOX, DO
43	*E. coli*	Human stool	AMP, AMC, CN, ATM, FEB, E, CRO, E, CIP, AX
	*P. mirabilis*		AMP, AMC, ATM, FEB, CRO, E, FOX, CIP, DO, SXT, AX
44	*E. coli*	Human stool	AMP, AMC, FEB, CRO, E, FOX, SXT, AX
45	*S.*Typhimurium	Human stool	AMP, AMC, ATM, FEB, CRO, E, FOX, CIP, SXT, AX
	*E. coli*		AMP, AMC, ATM, FEB, CRO, E, FOX, CIP, C, DO, SXT, AX
46	*S.*Typhimurium	Human stool	AMP, AMC, CN, TGC, ATM, FEB, CRO, E, FOX, CIP, C, SXT, AX
47	*S.*Typhimurium	Human stool	AMP, AMC, CN, AK, ATM, FEB, CRO, E, CIP, AX
	*E. coli*		AMP, AMC, CN, TGC, AK, IPM, ATM, FEB, CRO, E, FOX, CIP, C, DO, SXT, AX
48	*S.*Typhimurium	Human stool	AMP, AMC, CN, SAM, ATM, CRO, E, FOX, CIP, C, DO, SXT, AX

CN, gentamycin; AMP, ampicillin; AMC, amoxicillin-clavulanic acid; TGC, tigecycline; AK, amikacin; IPM, imipenem; SAM, ampicillin/sulbactam; ATM, aztreonam; FEB, cefepime; CRO, ceftriaxone; E, erythromycin; FOX, cefoxitin; CIP, ciprofloxacin; C, chloramphenicol; DO, doxycycline; SXT, trimethoprim/sulfamethaxole; AX, amoxicillin.

### Characterization of red kidney bean seeds phenolic-rich extract

The TPCs and TFCs of the methanolic extract obtained from red kidney bean seeds flour were determined. The TPCs of the extract were 53 ± 2 mg GAE g^–1^ extract. Meanwhile, the TFCs of the extract were 26 ± 1 mg QE g^–1^ extract.

**FIGURE 2:**
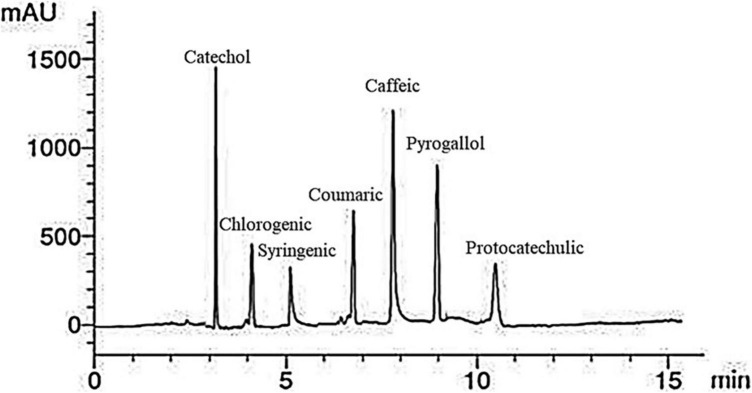
High-performance liquid chromatography-chromatogram of major phenolic compounds in red kidney bean seeds. mAU, milli-absorbance unit.

A representative HPLC phenolic compounds analysis chromatogram of red kidney bean methanolic extract is presented in Figure 2. Seven peaks of retention times at 3, 4, 5.1, 6.9, 8, 9, and 10.5 min dominated the chromatogram from the separation of red kidney bean methanolic extract. Table 4 listed the phenolic compounds contents (1–7) in the red kidney bean methanolic extract. The highest content is catechol (17.63 μg/mL) followed by caffeic (14.55 μg/mL), pyrogallol (13.12 μg/mL), coumaric (10.60 μg/mL), protocatechulic (6.33 μg/mL), chlorogenic (6.12 μg/mL), and syringenic (5.41 μg/mL).

**TABLE 4:** Major phenolic compounds in red kidney bean seeds estimated by HPLC.

Compounds	Retention time (min)	Concentration (μg/mL)
Catechol	3	17.63
Chlorogenic	4	6.12
Syringenic	5.1	5.41
Coumaric	6.9	10.60
Caffeic	8	14.55
Pyrogallol	9	13.12
Protocatechulic	10.5	6.33

**FIGURE 3:**
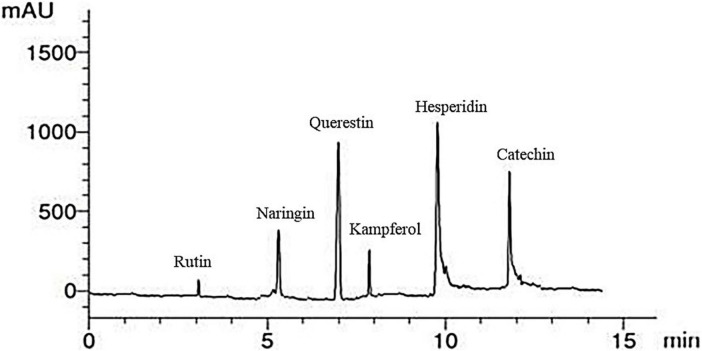
High-performance liquid chromatography-chromatogram of major flavonoid compounds in red kidney bean seeds. mAU, milli-absorbance unit.

An illustrative chromatogram of the HPLC flavonoid composites analysis of red kidney bean methanolic extract is publicized in Figure 3. Six peaks with retention times at 3, 5.3, 7, 8, 10, and 12.01 min dominated the chromatogram from the separation of red kidney bean methanolic extract. Table 5 demonstrates the phenolic compound contents (1–6) in the red kidney bean methanolic extract. The highest content is hesperidin (11.37 μg/mL) followed by catechin (10.41 μg/mL), quercetin (9.12 μg/mL), naringin (6.56 μg/mL), kaempferol (4.24 μg/mL), and rutin (1.56 μg/mL).

**TABLE 5:** Major flavonoid compounds in red kidney bean seeds estimated by HPLC.

Compounds	Retention time (min)	Concentration (μg/mL)
Rutin	3	1.56
Naringin	5.3	6.56
Quercetin	7	9.12
Kaempferol	8	4.24
Hesperidin	10	11.37
Catechin	12.01	10.41

### Protein characterization

**FIGURE 4:**
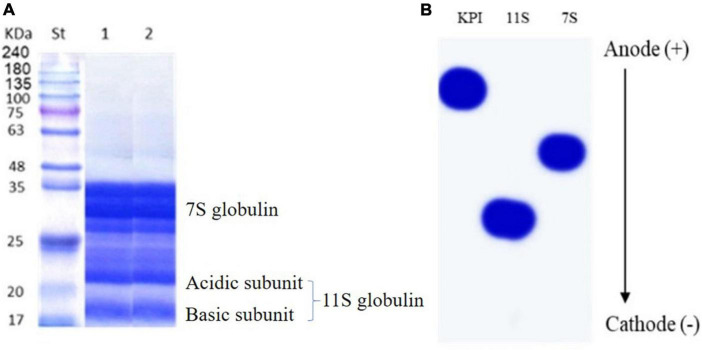
**(A)** SDS-PAGE of defatted red kidney bean seeds (lane 1) and red kidney bean protein isolate (lane 2) compared to protein marker (St). **(B)** Urea-PAGE red kidney bean protein isolate (KPI), 11S globulin, and 7S globulin.

Sodium dodecyl sulfate-polyacrylamide gel electrophoresis patterns of defatted red kidney bean seeds and red kidney beans protein isolate (KPI) are presented in Figure 4A. Both involved two major portions: 7S (vicilin) and 11S (legumin) globulins. The bands matching legumin are 19 (basic subunits) and 22 (acidic subunits) kDa. The bands conforming to vicilin are about 35, 33, and 28 kDa, indicating α/, α, and β subunits, respectively.

Figure 4B shows the urea-PAGE patterns for KPI, 7S, and 11S globulins. The passage in urea-PAGE of 7S and 11S globulins in the cathode direction was substantially faster than their corresponding KPI, representing more positive charges.

**FIGURE 5:**
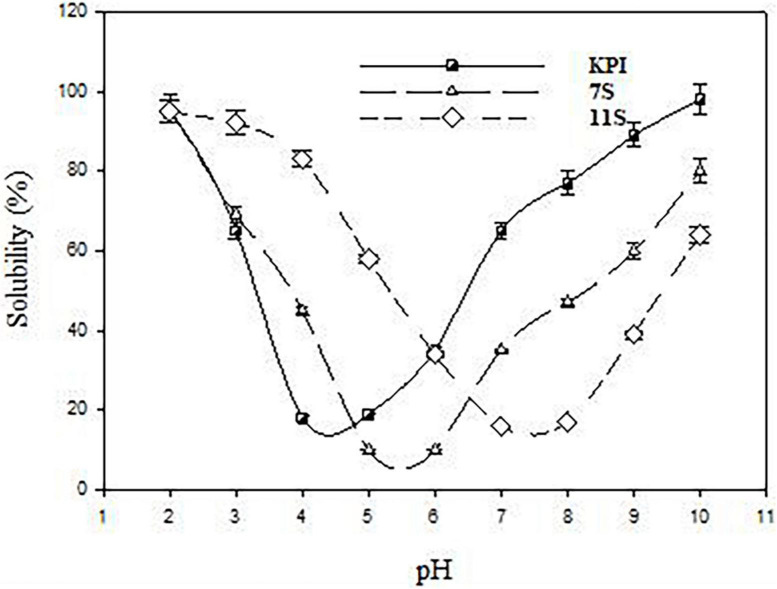
pH solubility profile of red kidney bean protein isolate (KPI), 7S globulin (7S), and 11S globulin (11S).

The pH solubility profile of KPI, 7S globulin (7S), and 11S globulin (11S) are presented in Figure 5. KPI, 7S, and 11S globulins have broad solubility minima at pH 4.5, 5.5, and 7.5, respectively (Figure 5).

**FIGURE 6:**
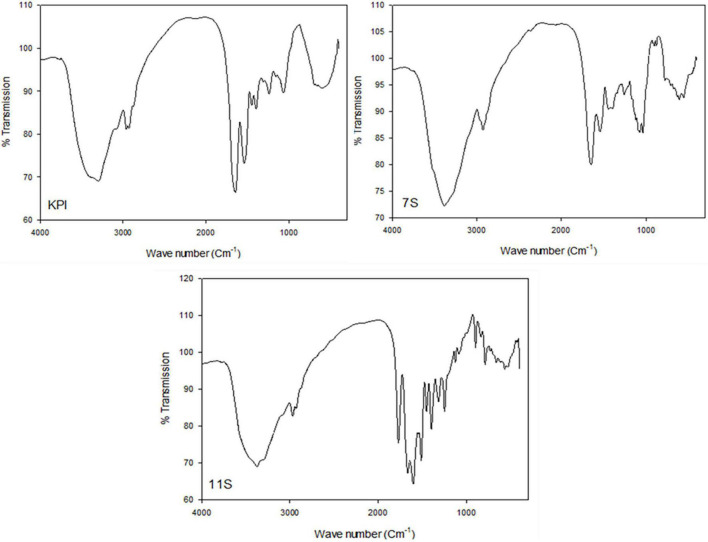
FTIR different spectra of red kidney bean protein isolate (KPI), 7S globulin (7S) and 11S globulin (11S).

Fourier transform infrared spectroscopy different spectra of KPI, 7S, and 11S globulins are presented in Figure 6. The amide-I region in the proteins IR spectra (1700–1600 cm^–1^) is best discernible from their secondary structure including their α-helix, β-sheet, turns, coils, etc. Thus, the amide-I area was assessed to estimate the proportion of various secondary structures in KPI, 7S, and 11S globulins. The primary amide I peak was observed at wave numbers 1636, 1634, and 1620 cm^–1^ for KPI, 7S, and 11S globulin, respectively.

### Antimicrobial susceptibilities of *Phaseolus vulgaris* against multidrug-resistant *Enterobacterales*

*Phaseolus vulgaris* extracts were examined against MDR *Enterobacterales* (*n* = 13) of high MAR indices (0.647–0.941) (Table 6). Our results revealed that the highest activity was reported for protein components in all concentrations (53.85%; inhibition zone diameter mean 17.14 ± 1.51 – 28.14 ± 1.48; MIC value = 0.125–2 μg/mL) followed by 30.77% for each of 7S globulin [inhibition zone diameter mean 16.75 ± 1.72 – 27.75 ± 1.59; MIC value = 0.5–2 μg/mL and 11S globulin (inhibition zone diameter mean 13.67 ± 1.76 – 24.33 ± 1.85; MIC value = 0.5-2 μg/mL)] and finally methanol (15.38%; inhibition zone diameter mean 10.50 ± 0.20 – 21.50 ± 0.20; MIC value = 2 μg/mL). Statistically, significant differences (*P* < 0.05) were reported between zone diameters of different concentrations of all antimicrobial agents for *P. mirabilis* isolates. Whereas, significant variations were observed for all antimicrobial agents except 11S globulin in tested *K. pneumoniae* isolates. Regarding *E. coli*, significant differences were noted only between different concentrations of 11S globulin and methanolic extract of RKP, while the antibacterial activity of the total protein components and 11S globulin differed statistically in examined *Salmonella* species. The MIC results of all antimicrobials differed statistically (*P* < 0.05) for tested *Enterobacterales* species except for *P. mirabilis* (*P* > 0.05).

**TABLE 6:** Antimicrobial activities of *Phaseolus vulgaris* extracts against *Enterobacterales* isolates.

Isolate no.	Code no.	Source	Species	MAR index	Antimicrobial agents																			
					Total protein components					7S Globulin					11S Globulin					Methanolic extract			
					Zone diameters/concentration for agar well diffusion method (mm)*				MIC μg/mL	Zone diameters/concentration for agar well diffusion method (mm)*				MIC μg/mL	Zone diameters/concentration for agar well diffusion method (mm)*				MIC μg/mL	Zone diameters/concentration for agar well diffusion method (mm)*				MIC μg/mL

					**100**	**75**	**50**	**25**		**100**	**75**	**50**	**25**		**100**	**75**	**50**	**25**		**100**	**75**	**50**	**25**	
1	32	Human stool	*E. coli*	0.941	17	13	8	7	128	0	0	0	0	128	9	6	2	0	128	8	4	3	0	128
2	31	Human stool	*E. coli*	0.823	34	30	27	24	0.125	35	32	28	22	0.5	21	18	14	10	1	9	3	2	0	64
3	47	Human stool	*E. coli*	0.941	17	13	10	7	64	2	0	0	0	128	10	7	4	2	128	9	6	3	2	128
Means ± SE					22.6 ± 5.4	18.6 ± 4.8	15.0 ± 6.0	12.6 ± 5.1		12.3 ± 3.5	10.6 ± 2.5	9.3 ± 2.4	7.3 ± 2.0		13.3 ± 3.8***^a^***	10.3 ± 3.3***^ab^***	6.6 ± 3.7***^ab^***	4.0 ± 1.1***^b^***		8.6 ± 0.3***^a^***	4.3 ± 0.8***^b^***	2.6 ± 0.1***^c^***	0.6 ± 0.1***^d^***	
4	1	Raw milk	*K. pneumoniae*	0.764	30	27	22	20	2	16	12	10	7	32	11	9	5	3	128	14	9	7	5	64
5	2	Raw milk	*K. pneumoniae*	0.705	33	30	25	21	0.5	17	13	10	6	16	32	29	24	21	0.5	13	11	6	5	64
6	5	Raw milk	*K. pneumoniae*	0.705	16	14	10	7	32	28	26	23	17	1	18	15	12	10	16	11	8	6	4	16
Means ± SE					26.3 ± 3.2***^a^***	23.7 ± 3.9***^ab^***	19.0 ± 3.6***^ab^***	16.0 ± 1.5***^b^***		20.3 ± 3.8***^a^***	17.0 ± 4.5***^ab^***	14.3 ± 4.3***^ab^***	10.0 ± 3.5***^b^***		20.3 ± 6.2	17.7 ± 5.9	13.7 ± 5.5	11.3 ± 5.2		12.7 ± 0.9***^a^***	9.3 ± 0.8***^a^***	6.3 ± 0.3***^b^***	4.7 ± 0.2***^c^***	
7	48	Human stool	*S.* Typhimurium	0.764	15	11	7	5	128	27	25	23	20	2	12	9	6	2	128	10	6	4	2	64
8	32	Human stool	*S.* Typhimurium	0.941	16	12	7	5	128	4	1	0	0	128	11	8	4	2	128	22	18	16	11	2
9	47	Human stool	*S.* Typhimurium	0.941	32	29	25	20	1	21	19	13	8	2	20	17	14	10	2	21	15	14	10	2
10	12	Meat product	*S.* Enteritidis	0.705	21	18	14	10	2	3	0	0	0	128	19	16	13	9	2	7	6	3	2	128
Means ± SE					21.0 ± 3.9***^a^***	17.5 ± 4.1***^ab^***	13.3 ± 4.3***^ab^***	10.0 ± 1.5***^b^***		13.8 ± 6.0	11.3 ± 6.3	9.0 ± 5.6	7.0 ± 3.7		15.5 ± 2.3***^a^***	12.5 ± 2.1***^a^***	9.3 ± 2.5***^ab^***	5.8 ± 1.8***^b^***		15.0 ± 6.8	11.3 ± 5.1	9.3 ± 5.4	6.3 ± 4.2	
11	43	Human stool	*P. mirabilis*	0.647	23	19	15	11	2	0	0	0	0	128	10	7	3	0	128	7	4	3	2	128
12	16	Fresh meat	*P. mirabilis*	1	24	20	17	14	1	0	0	0	0	128	15	11	9	6	64	4	0	0	0	128
13	39	Human stool	*P. mirabilis*	0.764	18	15	11	6	64	16	11	8	4	64	17	13	8	6	64	5	3	1	0	128
Means ± SE					21.7 ± 1.9***^a^***	18.0 ± 1.5***^a^***	14.3 ± 1.8***^ab^***	10.3 ± 2.3***^b^***		5.3 ± 0.6***^a^***	3.7 ± 0.5***^ab^***	2.7 ± 0.2***^ab^***	1.3 ± 0.2***^b^***		14.0 ± 2.1***^a^***	10.3 ± 1.8***^a^***	6.7 ± 1.9***^b^***	4.0 ± 1.1***^b^***		5.3 ± 0.7***^a^***	2.3 ± 0.4***^b^***	1.3 ± 0.1***^c^***	0.7 ± 0.1***^c^***	
Overall average ± SE					22.8 ± 2.0***^a^***	19.3 ± 2.2***^ab^***	15.2 ± 2.0***^bc^***	12.1 ± 1.8***^c^***		13.0 ± 3.4	10.7 ± 3.2	8.8 ± 2.8	6.5 ± 3.3		15.8 ± 1.8***^a^***	12.7 ± 1.8***^ab^***	9.1 ± 1.7***^bc^***	6.2 ± 1.6***^c^***		10.8 ± 1.5***^a^***	7.2 ± 1.4***^ab^***	5.2 ± 1.3***^b^***	3.3 ± 1.1***^b^***	

Isolates code numbers are those deposited in Table 3. ^a–c^Means with different superscripts in the same column within each variable are significantly different (*P < 0.05).

## Discussion

Antimicrobial-resistant bacteria denote a significant concern influencing veterinary medicine and public health (Adams et al., 2018). It is crucial to evaluate the efficacy of antimicrobial stewardship programs as well as practices of infection prevention and control, especially in critically diseased patients (Alkofide et al., 2020). This study was designed to explore the antimicrobial resistance of *Enterobacterales* recovered from animal and human origins as well as alternative approaches for resistance mitigation.

Herein, *Enterobacterales* were recovered from 38.75% of analyzed samples from animal sources. Although milk is high-quality nutritional food, it is considered a medium for the growth, multiplication, and transfer of many microbes to humans (Donkor et al., 2007).

The percentage of *Enterobacterales* in raw milk was 60%, which was almost similar to what was conveyed previously (36%) (Tartor et al., 2021), higher than that reported by El-Mokadem et al. (2020) (14.28%) but lower than that obtained by a previous study (Sobeiha et al., 2020) in Egypt (84%). However, the percentage of *Enterobacterales* in milk products was 29.4%, whereas Canizalez-Roman et al. (2013) reported a nearly similar existence of diarrheic *E. coli* in dairy products (28.4%). Bacterial contamination of milk and its products could be retrieved from the udder and/or milk handling equipment (Oliver et al., 2005). *E. coli* recovery from milk indicates fecal contamination and the existence of toxigenic and/or enteropathogenic bacteria, both of which pose public health risks (Ali and Abdelgadir, 2011). These results suggest a need for improvement of milking operations and good manufacturing practices (Costanzo et al., 2020).

A higher probability of microbial contamination of meat samples may be from the intestinal tract or certain animal illnesses resulting in gastrointestinal ruptures that lead to cross-contamination (Gwida et al., 2014). Coliforms can be found in a diversity of environments, including water, soil, and vegetation and are commonly used as an indicator of the hygienic feature of foods. They can be found profusely in the feces of warm-blooded animals. Their existence could indicate other pathogenic microorganisms of fecal origin (Zhao et al., 2001). This is a substantial public health issue around the world, with significant economic implications in developed countries (Abdelmalek et al., 2019). In the present work, the existence of *Enterobacterales* in meat and its products was 25.5%. This aligns with previous research (Enayat et al., 2012; Elhawary et al., 2016) in which *Enterobacterales* isolates were detected in meat samples with percentages of 67 and 54%, respectively. However, Abd El-Aziz et al. (2018) reported *Enterobacterales* in 80.33% of examined meat samples. The variations in the results obtained by different investigators may be due to differences in handling, manufacturing practices, and the time of exposure.

In the current study, *Enterobacterales* isolates were recovered from human stools with a percentage of 56.6%, with a predominance of *E. coli* (40%) either single or mixed with *Salmonella* species, whereas no *Klebsiella* species were isolated. Previously, a lower prevalence of *Enterobacterales* (10.8%) among patients diagnosed with gastrointestinal infection was recorded (Rustam et al., 2006). Meanwhile, 22.7% of human stools were positive for *K. pneumonia* lately in Egypt (Elmonir et al., 2021). However, *Salmonella* infections were previously reported in human stools; 4% (Abd El-Aziz et al., 2021) and 44% (Kasumba et al., 2021). A negative stool culture may be attributed to the reason that *Enterobacterales* isolates were not present in sufficient numbers to be detected. Moreover, Tarr et al. (2005) documented that enterohemorrhagic *E. coli* are difficult to be identified in human stools in late infection and the recovery rate decreased to 33.3% in stools collected more than 6 days after the outset of diarrhea.

Infections occurred by antibiotic-resistant Gram-negatives, mainly those developed in hospitals, pose serious threat to global public health (Exner et al., 2017; Elmowalid et al., 2018; Abd El-Aziz et al., 2020; Elmonir et al., 2021). Antimicrobial resistance represents a global crisis that endangers the efficacy of antibiotics and consequently the efficiency of treatment provided. The mortality rate associated with drug resistance is substantial, adding to the burden of infectious diseases (Livermore, 2009; De Kraker et al., 2011).

In this study, among 57 tested *Enterobacterales* isolates from both human and animal sources, antimicrobial resistance was mostly observed for amoxicillin-clavulanate (100%), ampicillin (75.44%), erythromycin (71.93%), cefoxitin (70.18%), amoxicillin (66.66%), ceftriaxone (64.91%), and trimethoprim/sulfamethoxazole (56.14%). However, a moderate sensitivity level was observed for chloramphenicol (47.37%), followed by ciprofloxacin and tigecycline (40.35 and 42.11%, respectively). In previous reports, high resistance levels were detected for the abovementioned antimicrobial agents (Abd El-Aziz and Gharib, 2015; Elmonir et al., 2021; Kotb et al., 2022).

Kidney beans‘ health advantages are attributable to their particular phytochemicals and nutrients. Kidney beans are rich in protein, dietary fiber, minerals, carbohydrates, and phytochemicals, containing phenolic compounds (Kan et al., 2017). Bean phenolic antioxidants have been demonstrated to affect biomarkers linked to health and help to prevent chronic diseases (Redan et al., 2013). The anti-inflammatory, antioxidant, anti-atherosclerotic, anticancer, antihypertensive, and antiaging properties of phenolic chemicals found in kidney beans have been demonstrated (García-Lafuente et al., 2014). Polyphenolic chemicals generated from plant tissue have been shown to offer health benefits in several studies (Aboghoniem et al., 2022; Darwesh et al., 2022). This study proved that red kidney bean polyphenols had antibacterial properties against *Enterobacterales*. The highest activity was reported for protein components in all concentrations (53.85%; inhibition zone diameter mean 17.14 ± 1.51–28.14 ± 1.48; MIC value = 0.125–2 μg/mL) followed by 7S globulin and 11S globulin (30.77% each) and finally methanol (15.38%). These findings demonstrate new visions into the antimicrobial potential of red kidney beans and propose an auspicious therapeutic action against MDR *Enterobacterales*, while no available data document this investigation.

## Conclusion

This is a comprehensive study on the antibacterial action of red kidney beans (*Phaseolus vulgaris* L.) against *Enterobacterales* isolated from human and animal sources. The overall occurrence rate of *Enterobacterales* significantly differed between animal and human sources. Antimicrobial susceptibility results declared that *Enterobacterales* isolates were highly resistant to different broad-spectrum antibiotics. The protein component of red kidney bean seeds was the most effective extract, followed by 7S and 11S globulin, exhibiting their potential as promising alternatives for improving future dosing strategies against *Enterobacterales* as a first report.

## Data availability statement

The datasets presented in this study can be found in online repositories. The names of the repository/repositories and accession number(s) can be found in the article/Supplementary material.

## Ethics statement

Ethical approval was not provided for this study on human participants because the study was conducted following the Ethics of the World Medical Association (Declaration of Helsinki). Written informed consent was obtained from the patients for participation in this study. We collected human stool samples for the study. The patients/participants provided their written informed consent to participate in this study.

## Author contributions

AE and NKA contributed similarly to the study’s design and conception. WH, DHMA, DE-H, and EE conceived the work and contributed to the design. AE, NKA, EE, WH, DHMA, DE-H, and AO participated in the data interpretation. AE, NKA, and AO typed the draft of the manuscript. All authors revised the manuscript and approved the last version.
