# Supplementary figures and images for: Antibacterial activity of bioactive compounds extracted from red kidney bean (Phaseolus vulgaris L.) seeds against multidrug-resistant Enterobacterales
Source: Front Microbiol. 2022 Nov 7;13:1035586. doi: 10.3389/fmicb.2022.1035586 (PMC9676267; doi:10.3389/fmicb.2022.1035586)

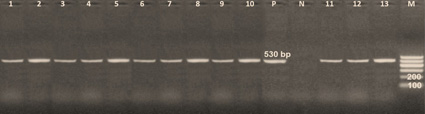

Supplement: Supplementary file 1 [file Image_1.JPEG]

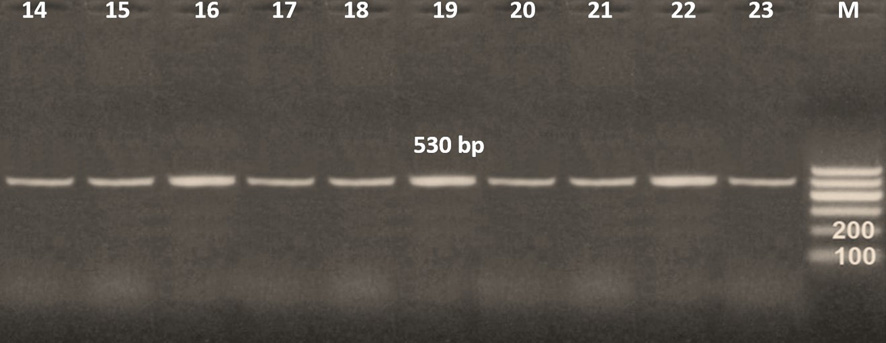

Supplement: Supplementary file 2 [file Image_2.JPEG]

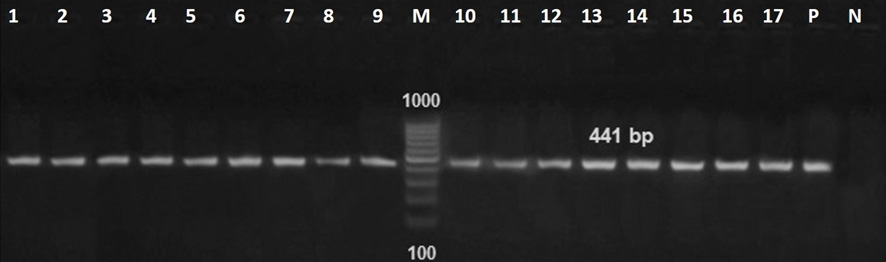

Supplement: Supplementary file 3 [file Image_3.JPEG]

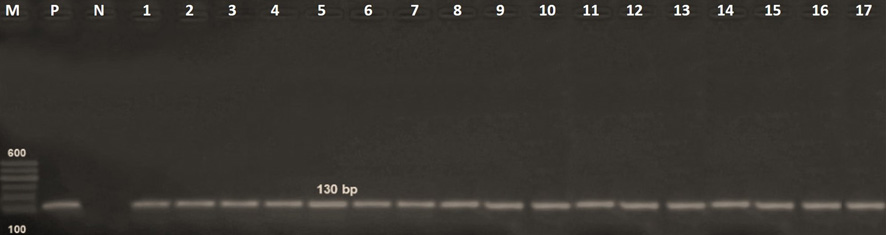

Supplement: Supplementary file 4 [file Image_4.JPEG]

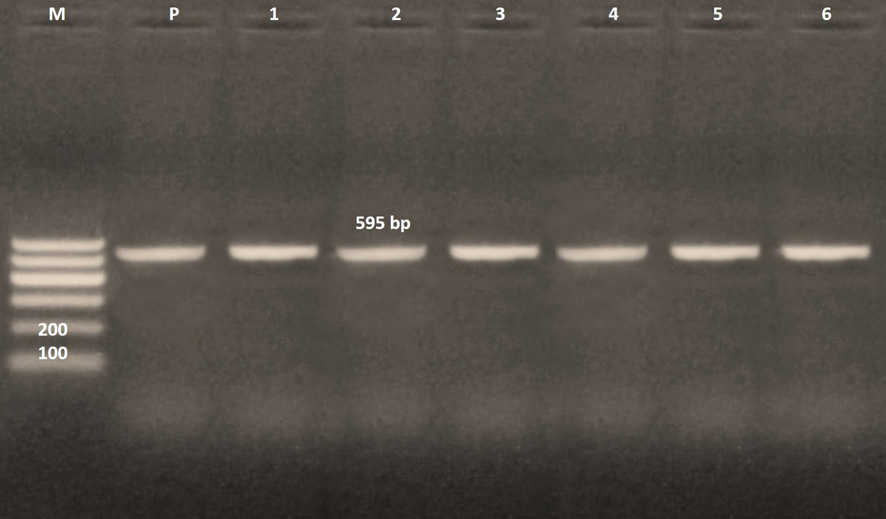

Supplement: Supplementary file 5 [file Image_5.JPEG]

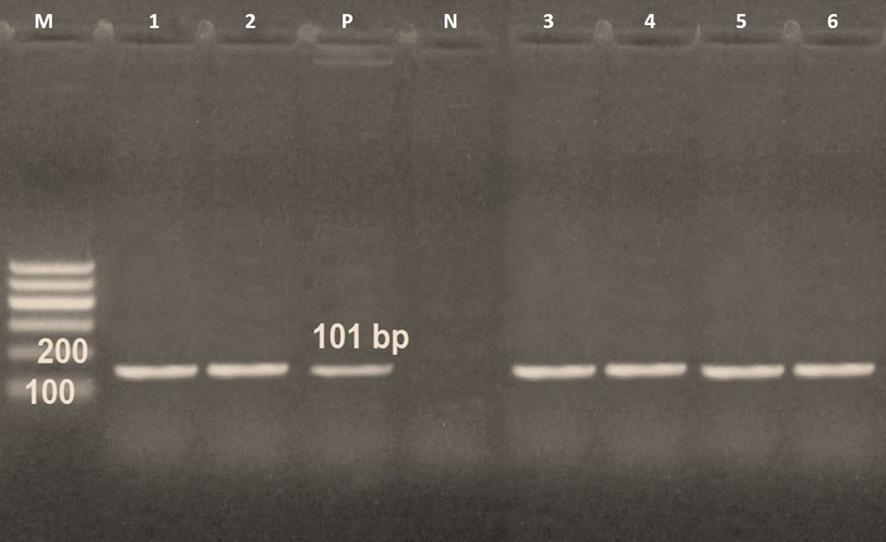

Supplement: Supplementary file 6 [file Image_6.JPEG]

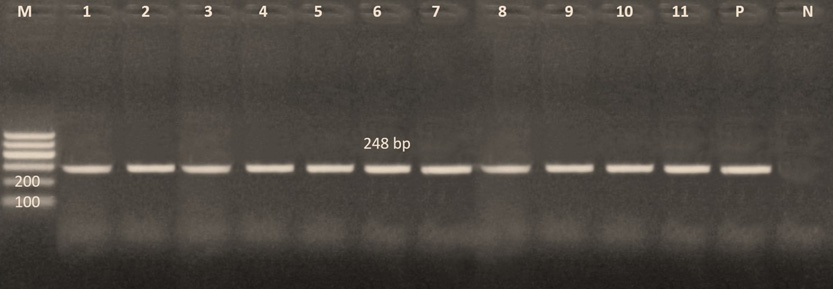

Supplement: Supplementary file 7 [file Image_7.JPEG]

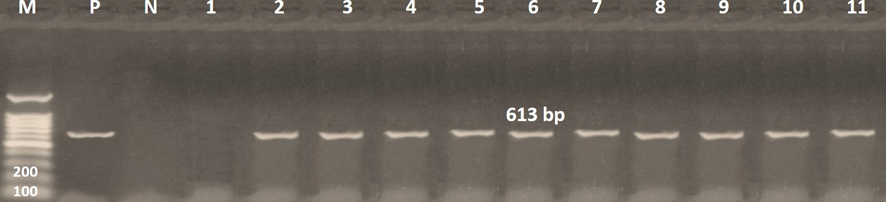

Supplement: Supplementary file 8 [file Image_8.JPEG]

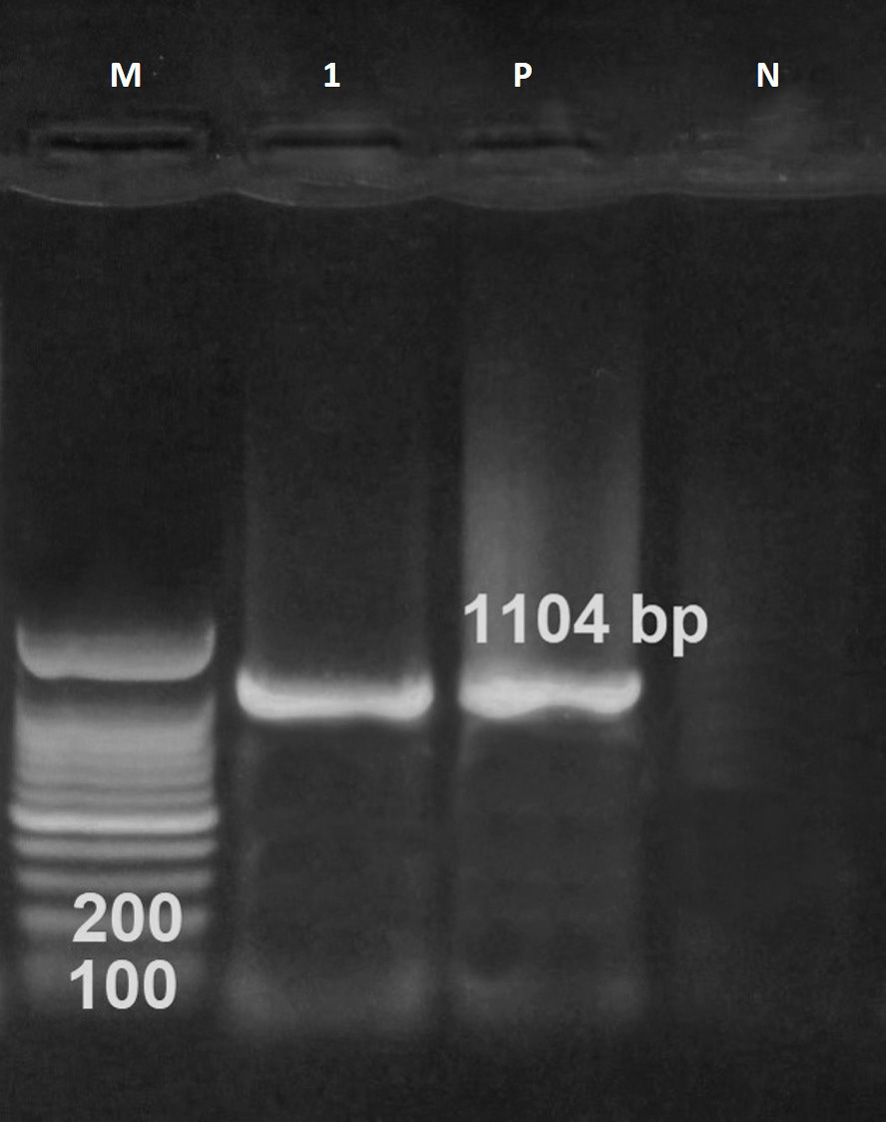

Supplement: Supplementary file 9 [file Image_9.JPEG]
