# Supplementary material for: Antibacterial activity of bioactive compounds extracted from red kidney bean (Phaseolus vulgaris L.) seeds against multidrug-resistant Enterobacterales
Source: Front Microbiol. 2022 Nov 7;13:1035586. doi: 10.3389/fmicb.2022.1035586 (PMC9676267; doi:10.3389/fmicb.2022.1035586)
Supplement: Supplementary file 10 [file Data_Sheet_1.docx]

**Supplementary Figure 1:** PCR amplification of *E. coli* isolates using species-specific primer pairs for *uidA* gene at 530 bp. Lane M: 100 bp molecular weight marker, lanes 1-23: positive *E. coli* isolates. Lane P: positive control, lane N: negative control.

**Supplementary Figure 2:** Agarose gel electrophoresis showing PCR amplification fragments for genus-specific *gyrA* (441 bp) (A) and species-specific *16S-23S ITS* (130 bp) (B) genes. Lane M: 100 bp molecular weight marker, lanes 1-17: positive *K. pneumoniae* isolates. Lane P: positive control, lane N: negative control.

**Supplementary Figure 3:** Agarose gel electrophoresis exhibiting PCR amplification products for genus-specific *atpD* (595 bp) (A) and species-specific *ureR* (101 bp) (B) genes. Lane M: 100 bp molecular weight marker, lanes 1-6: positive *P. mirabilis* isolates. Lane P: positive control, lane N: negative control.

**Supplementary Figure 4:** Agarose gel electrophoresis showing PCR amplicons of *Salmonella* genus-specific *invA* (441 bp) gene (A; Lanes 1-11), *S.* Typhimurium species-specific *fliC* (613 bp) gene (B; Lanes 2-11), and *S.* Enteritidis *sef* (1104 bp) gene (C; Lane 1). Lane M: 100 bp molecular weight marker, lane P: positive control, lane N: negative control.
