# Supplementary material for: Antibacterial activity of bioactive compounds extracted from red kidney bean (Phaseolus vulgaris L.) seeds against multidrug-resistant Enterobacterales
Source: Front Microbiol. 2022 Nov 7;13:1035586. doi: 10.3389/fmicb.2022.1035586 (PMC9676267; doi:10.3389/fmicb.2022.1035586)
Supplement: Supplementary file 11 [file Table_1.DOCX]

**Supplementary Table 1:** Oligonucleotide primers used in this study

| **Bacterial strain** | **Gene** | **Oligonucleotide sequence (5′ → 3′)** | **Amplicon size (bp)** | **Reference** |
| --- | --- | --- | --- | --- |
| *E. coli* | *uidA* | F: GTCACGCCGTATGTTATTG  R: CCAAAGCCAGTAAAGTAGAAC | 530 | (Litty et al., 2013) |
| *Klebsiella* species | *gyrA* | F: CGCGTACTATACGCCATGAACGTA  R: ACCGTTGATCACTTCGGTCAGG | 441 | (Brisse, and Verhoef 2001) |
| *K. pneumoniae* | *16S-23SITS* | F: ATTTGAAGAGGTTGCAAACGAT  R: TTCACTCTGAAGTTTTCTTGTGTTC | 130 | (Turton et al., 2010) |
| *Proteus* species | *atpD* | F: GTATCATGAACGTTCTGGGTAC  R: TGAAGTGATACGCTCTTGCAG | 595 | (Bi et al., 2013) |
| *Proteus* *mirabilis* | *ureR* | F: GGAAACGGTGGCTAATACCGCATAAT  R: GCAGCGCTAGGTGAGCCTAATGGG | 101 | (Zhang et al., 2013) |
| *Salmonella* species | *invA* | F: GTGAAATTATCGCCACGTTCGGGCAA  R: TCATCGCACCGTCAAAGGAACC | 248 | (Kumar et al., 2008) |
| *Salmonella* Typhimurium | *fliC* | F: CGGTGTTGCCCAGGTTGGTAAT  R: ACTGGTAAAGATGGCT | 613 | (Halimi et al., 2014) |
| *Salmonella* Enteritidis | *sef* | F: GCGAAAACCAATGCGACTGTA  R: CCCACCAGAAACATTCATCCC | 1104 | (Murugkar et al., 2003) |

bp, base pair
